# The inadequate corpus luteum

**DOI:** 10.1530/RAF-20-0044

**Published:** 2021-02-26

**Authors:** W Colin Duncan

**Affiliations:** 1 MRC Centre for Reproductive Health, The University of Edinburgh, Queen’s Medical Research Institute, Edinburgh, UK

**Keywords:** luteal phase defect, progesterone, infertility, miscarriage, luteal support

## Abstract

The corpus luteum is the source of progesterone in the luteal phase of the cycle and the initial two-thirds of the first trimester of pregnancy. Normal luteal function is required for fertility and the maintenance of pregnancy. Progesterone administration is increasingly used during fertility treatments and in early pregnancy to mitigate potentially inadequate corpus luteum function. This commentary considers the concept of the inadequate corpus luteum and the role and effects of exogenous progesterone. Progesterone supplementation does have important beneficial effects but we should be wary of therapeutic administration beyond or outside the evidence base.

**Lay summary:**

After an egg is released a structure is formed on the ovary called a corpus luteum (CL). This produces a huge amount of a hormone called progesterone. Progesterone makes the womb ready for pregnancy but if a pregnancy does not happen the CL disappears after 12–14 days and this causes a period. If a pregnancy occurs, then the pregnancy hormone (hCG) keeps the CL alive and its progesterone supports the pregnancy for the next 6–8 weeks until the placenta takes over and the corpus luteum disappears. That means that if the CL is not working correctly there could be problems getting pregnant or staying pregnant. If a CL is not producing enough progesterone it usually means there is a problem with the growing or releasing of the egg and treatment should focus on these areas. In IVF cycles, where normal hormones are switched off, the CL does not produce quite enough progesterone before the pregnancy test and extra progesterone is needed at this time. In recurrent or threatened miscarriage, however, there is not any evidence that the CL is not working well or progesterone is low. However, there is benefit in taking extra progesterone if there is bleeding in early pregnancy in women with previous miscarriages. This might be because of the effects of high-dose progesterone on the womb or immune system. As changes to the hormone environment in pregnancy may have some life-long consequences for the offspring we have to be careful only to give extra progesterone when we are sure it is needed.

The corpus luteum, formed from the cells of the dominant follicle after ovulation, is fundamental for human fertility. The progesterone it produces is absolutely required to prepare the endometrium for implantation and to maintain an early pregnancy (Duncan 2017). In the absence of a corpus luteum, the endometrium can be prepared for implantation (Critchley *et al.* 1990) and an early pregnancy can be maintained using exogenous progesterone (Tavaniotou *et al.* 2000). That would imply that if the corpus luteum was inadequate, and producing a suboptimal amount of progesterone, supplementation with progesterone would increase fertility and help maintain a pregnancy. Therefore, it is fundamentally important to determine if corpus luteum function can be inadequate and why that would happen?

## How the corpus luteum works

The corpus luteum is absolutely dependent on ligand stimulation of membrane LHCGR, by luteinising hormone (LH) or human chorionic gonadotrophin (hCG), in three ways. The first is its formation. It is the surge of LH at ovulation that is responsible for luteinisation of the follicular granulosa cells. They become terminally differentiated, unable to divide further, undergo hypertrophy, start to express the enzymatic machinery to be able to synthesise progesterone and develop lipid droplets in their cytoplasm, giving them a golden yellow colour. The second is its maintenance as luteal progesterone production is absolutely dependent on LHCGR stimulation. Removal of LH in the luteal phase by the withdrawal of gonadotrophin-releasing hormone (GnRH) (Hutchison & Zeleznik 1984) or by using a GnRH antagonist (Fraser *et al.* 1986) causes progesterone concentrations to plummet. The third is during maternal recognition of pregnancy where hCG from the implanting blastocyst, in exponentially increasing concentrations, rescues the corpus luteum from luteolysis to maintain luteal progesterone until the luteoplacental shift around 8–9 weeks of gestation (Duncan 2017).

## How much progesterone is enough?

**Figure 1:**
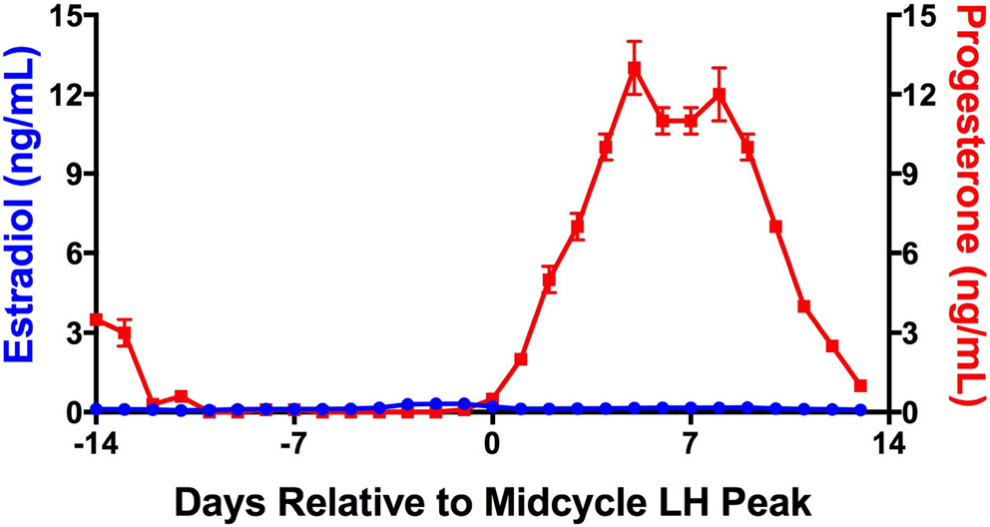
Hormone profile of the menstrual cycle of women. Concentrations of oestradiol and progesterone plotted on the same scale highlighting the dominance of progesterone. Data adapted from Groome *et al.* (1996), Duncan (2017).

It is fascinating to look at follicular oestradiol production and luteal progesterone production on the same scale (Fig. 1). Progesterone is the hormone that dominates the menstrual cycle. Progesterone concentrations above 25 nmol/L (7.7 ng/mL) are used in clinical practice to confirm ovulation. In a detailed monitored natural cycle, the average mid-luteal progesterone was 41.3 ± 3.2 nmol/L (13.0 ± 1.0 ng/mL) (Groome *et al.* 1996) although normal pregnancies have been reported at less than half this level (Yovich *et al.* 2015). In women undergoing gonadotrophin ovulation induction, the average timed mid-luteal progesterone concentrations in mono-ovulatory cycles was 49.6 nmol/L (15.6 ng/mL) with pregnancies reported at half this level (Arce *et al.* 2011).

In a study of endometrial secretory changes in the luteal phase, there was no evidence that infertile women had less decidualisation or more out of phase biopsies than fertile women. Indeed, there was significantly more abnormal luteal phase biopsies in the fertile population (Filicori *et al.* 1984). In a study looking at artificial cycles, where intramuscular progesterone was used, low-dose progesterone that increased serum progesterone to 17.5 ± 3.5 nmol/L (5.5 ±1.1 mg/mL) was just as able to create normal secretory endometrial changes as high-dose progesterone (61.1 ± 21 nmol/L; 19.2 ± 6.6 ng/mL). The corpus luteum is a robust endocrine gland that produces more progesterone than is required for fertility. That makes sense as mutations or genetic variants that absolutely prevent conception are evolutionary dead ends and heavily selected against.

## The inadequate corpus luteum in a natural cycle

It is not uncommon, however, in the infertility clinic, for women to present with suboptimal mid-luteal phase progesterone concentrations (Wathen *et al.* 1984) or a shorter duration of the luteal phase. It is therefore clear that the corpus luteum can be inadequate and there are three possibilities to explain why this might happen. The first is that there is an intrinsic cellular defect in luteal steroidogenic function or vascularisation. For example, the use of VEGF blockade during luteal development reduces luteal vascularisation and markedly reduces progesterone concentrations (Fraser & Duncan 2009). If the problem was purely at the level of the corpus luteum it would imply that progesterone supplementation in women with low progesterone during a natural cycle would improve fertility. There is no evidence that progesterone supplementation in natural cycles improves fertility and most would agree that there is no intrinsic pathology of the corpus luteum that is associated with reduced fertility in women (Shivapathasundram *et al.* 2011).

The second possibility is that it relates to an inadequate or premature LH surge meaning that the formation of the corpus luteum was suboptimal. As the LH surge is required for oocyte activation suboptimal luteal function may be a marker of impaired oocyte maturation. Women who run with higher LH, such as those with premature ovarian insufficiency or those with polycystic ovary syndrome (PCOS) are less likely to be able to generate a significant LH surge, with an appropriate area under the curve, and are more likely to have premature luteinisation (Goh & Ratnam 1990). In addition, women taking anti-oestrogen fertility drugs, such as clomifene or letrozole, in the early follicular phase may have reduced capacity for oestrogen-regulated positive feedback to generate the LH surge, particularly if follicular growth is rapid. Those who run with low LH, such as women with hypogonadotrophic hypogonadism or hyperprolactinaemia, are also less able to generate an adequate LH surge. Indeed, the classic inadequate corpus luteum is present in women who are breastfeeding (McNeilly *et al.* 1982). There is good evidence that a suboptimal LH surge would be associated with an inadequate corpus luteum.

The third possibility is where the inadequate corpus luteum is a result of a substandard pre-ovulatory follicle. Poor follicular growth is also associated with a reduction in oocyte quality. A follicle with suboptimal growth, or one seeing a premature LH surge, is not a suitable environment for normal oocyte development and would not form a normally functioning corpus luteum. This may be a result of ovarian ageing, naturally declining fertility or poor follicular reserve and as such it difficult to treat. However, where there are hormonal reasons for poor follicular development, such as PCOS or hypogonadotrophic hypogonadism ovulation induction strategies focussing on the follicle would help optimise follicular growth, oocyte development and luteal function.

Overall an inadequate corpus luteum in a normal cycle is a marker of problems before and during ovulation. As such it reflects suboptimal oocyte development. That means the focus should not be on supplementation of progesterone but rather on mechanisms to improve oocyte and luteal quality using strategies to improve follicular growth, using ovulation induction, or the LH surge, using ovulatory hCG injection. This is supported by a study of the inadequate corpus luteum in normally cycling women where there was lower oestrogen in the follicular phase and reduced LH concentrations during the LH surge (Schliep *et al.* 2014). In a natural cycle management of an inadequate luteal phase should target the follicular and ovulatory phases of the cycle.

## The inadequate corpus luteum in assisted conception

**Figure 2:**
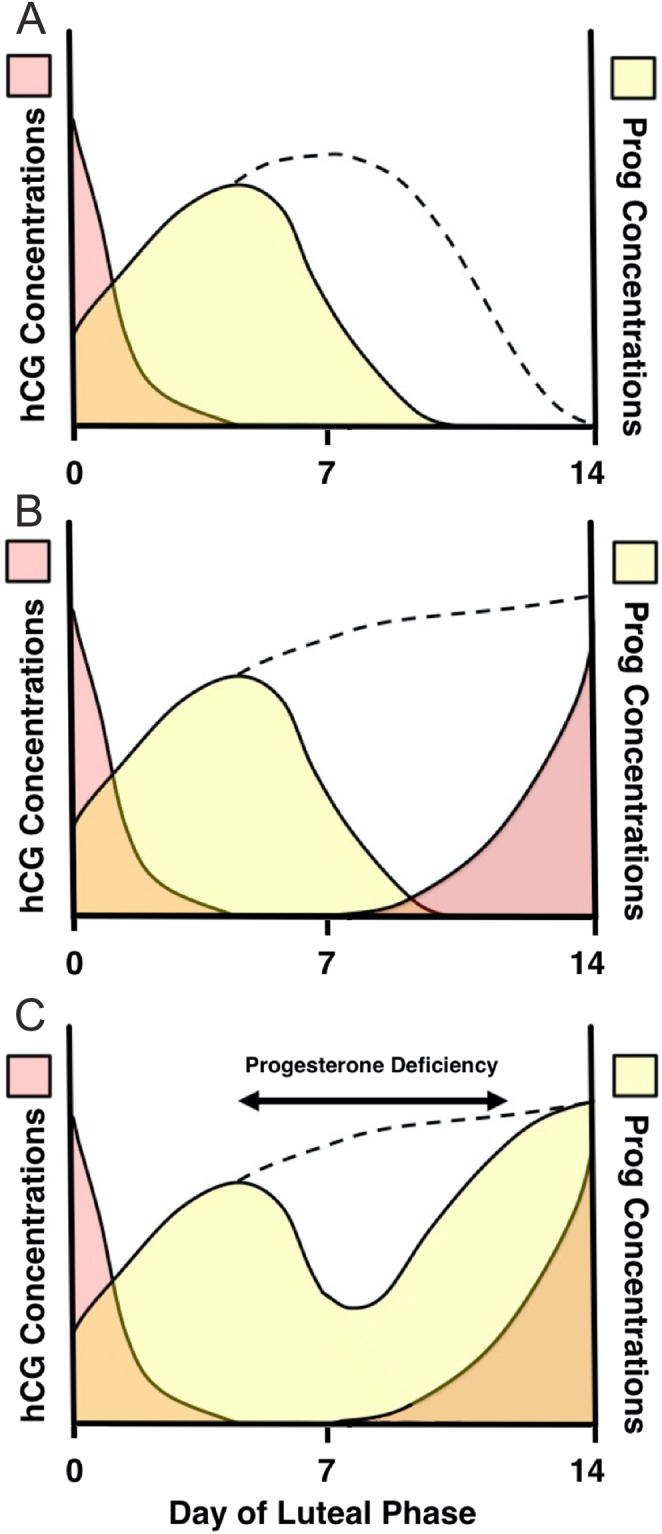
The requirement for luteal support during assisted conception. (A) In a downregulated cycle, exogenous hCG induces progesterone production, but in the absence of LH progesterone output declines earlier than during a natural cycle (dotted line). (B) In a conception cycle, endogenous hCG rises exponentially from LH+7 to maintain progesterone output (dotted line). (C) The endogenous hCG will rescue the corpus luteum in a downregulated cycle in assisted conception to maintain progesterone but there is a time of relative progesterone deficiency in the mid-luteal phase (Duncan 2017).

Ovarian stimulation regimes during assisted conception involve the use of GnRH agonists or GnRH antagonists to block pituitary LH secretion and prevent a premature LH surge. In the luteal phase of a downregulated cycle there is a deficiency of LH and thus suboptimal luteal function. When hCG is used to induce ovulation its long half-life (>24 h) means that early luteal progesterone production is normal but it is not enough to support a full luteal phase (Fig. 2). However, in a conception cycle, the endogenous hCG will rescue luteal progesterone production (Fig. 2). There is a stimulation deficit between the exogenous and endogenous hCG, which is not enough to trigger luteolysis, where progesterone secretion is inadequate.

In a GnRH agonist triggered cycle the short half-life of LH (20 min) means that the luteal phase will be even shorter and luteolysis would begin before the corpus luteum could be rescued by endogenous hCG. Luteal physiology would suggest that in an hCG-triggered down-regulated cycle progesterone production around the mid-luteal phase would be relatively deficient and supplementation with either progesterone or hCG would be required at this time (Fig. 2). However, in a cycle with a GnRH agonist trigger to induce an LH surge, in the absence of subsequent low-dose hCG administration (Kol 2019), progesterone supplementation would be required until the luteoplacental shift.

Meta-analysis of assisted conception cycles shows that luteal support after oocyte retrieval improves pregnancy rates (van der Linden *et al.* 2015). As the reduced progesterone is a consequence of reduced trophic hormone, post-ovulatory hCG injections OR 1.76 (1.08–2.86) improves pregnancy rates. However, hCG stimulates VEGF secretion, which is implicated as the key molecule in the development of ovarian hyperstimulation syndrome (OHSS). Progesterone supplementation also improves pregnancy rates OR 1.77 (1.09–2.81) (van der Linden *et al.* 2015) and as this does not stimulate VEGF the risk of OHSS is lower when compared to luteal support with hCG, OR 0.46 (0.30–0.71) (van der Linden *et al.* 2015). Thus, it is standard practice to use progesterone gels, pessaries or injections for post-oocyte collection luteal support.

As progesterone is stimulated by the artificial LH surge, it makes sense that progesterone support should start after rather than before this surge. Pregnancy rates are better if progesterone is started at the time of oocyte retrieval rather than the day before (Connell *et al.* 2015). As hCG will stimulate progesterone for several days after administration there is no difference, in pregnancy rate, if starting on day 2 or 3 after the oocyte collection rather than starting on the day of oocyte collection, while starting after this time resulted in a 16% decrease in pregnancy rate (Connell *et al.* 2015).

There is no change in pregnancy rate or pregnancy outcome if luteal support is discontinued after 2 weeks (Liu *et al.* 2012). Once endogenous hCG is present progesterone support is not required as the corpora lutea will be producing enough progesterone (Connell *et al.* 2015). However, clinical practice has not followed the evidence as in one survey progesterone support was continued until 10–12 weeks gestation in 67% cycles, when the foetal heartbeat was detected in 22% of cycles and discontinued at pregnancy test in only 12% of cycles (Liu *et al.* 2012). In another survey 40% of units continued progesterone support until 12 weeks’ gestation (Russell *et al.* 2015).

There is some controversy about progesterone support in frozen embryo replacement in natural cycles. Basic luteal physiology would suggest that this is not necessary. Indeed progesterone supplementation in natural frozen embryo replacement cycles did not increase pregnancy rates (Eftekhar *et al.* 2013). However, another study suggested a modest increase in pregnancy rates (Bjuresten *et al.* 2011). It is unclear whether this might reflect the common practice of using an hCG trigger in advance of the natural LH surge. A large randomised study is now trying to address this question (Saupstad *et al.* 2019).

There is an inadequate corpus luteum in down-regulated cycles and progesterone supplementation is required. However, there is no evidence that it is required after a positive pregnancy test in cycles where corpora lutea are present.

## The inadequate corpus luteum and first-trimester miscarriage

As luteal progesterone is absolutely required to support an early pregnancy until the luteoplacental shift, and removal of progesterone induces a miscarriage (Csapo & Pulkkinen 1978), inadequate progesterone in early pregnancy would increase the risk of miscarriage and progesterone supplementation would mitigate this risk. There is, however, no evidence for low endogenous progesterone causing miscarriage. The carefully timed mid-luteal progesterone in 192 women undergoing ovulation induction with hCG trigger who went on to a full-term singleton delivery was 25.85 ± 10.08 ng/mL while those having a miscarriage was 28.64 ± 16.96 ng/mL (Sallam *et al.* 1999). However, serum progesterone concentrations are lower in pregnancies that miscarry than in viable pregnancies (Szekeres-Bartho & Balasch 2008). The hCG dynamics of a pregnancy that will miscarry are often abnormal with low progesterone concentrations a consequence of suboptimal hCG increases, in a failing pregnancy, rather than a direct cause of miscarriage.

Although 15–20% of early pregnancies will miscarry in the first trimester the majority of these have chromosomal or morphological abnormalities and progesterone supplementation would have no benefit (Chetty & Duncan 2018). Around 1% of couples suffer from recurrent pregnancy loss, here defined as three or more miscarriages. As this is higher than chance alone, there must be some underlying causes that predispose couples to miscarriage (Chetty & Duncan 2018). While there may be genetic, structural or immunological causes most couples with recurrent miscarriage have no defined cause identified. Importantly, there is no reduction in luteal progesterone concentrations in women suffering from recurrent miscarriage (Ogasawara *et al.* 1997).

It may be that progesterone action is suboptimal in the presence of normal progesterone concentrations as it has been reported that decidualisation may be abnormal, with endometrial asynchrony, in women with recurrent miscarriage (Szekeres-Bartho & Balasch 2008) suggesting an endometrial resistance to progesterone. However, similar findings have been reported in fertile women (Filicori *et al.* 1984, Coutifaris *et al.* 2004) and subfertile women with endometriosis (Bulun *et al.* 2006) or implantation failure (Timeva *et al.* 2014). Recurrent pregnancy loss is not usually associated with subfertility. It is difficult to argue that a measurable progesterone deficit directly causes miscarriage.

Women with threatened miscarriage, and those with recurrent miscarriage, are often keen for treatment, and the use of progesterone supplementation to prevent miscarriage in early pregnancy is very common. The use of progesterone as a treatment in early pregnancy continues past the luteoplacental shift, when the luteal progesterone is minimal. The PROMISE study, a large placebo-controlled trial looking at progesterone supplementation in women with recurrent pregnancy loss, from the positive pregnancy test until 12 weeks of gestation, did not show evidence of efficacy for progesterone supplementation (Coomarasamy *et al.* 2015). Reassuringly, however, women with six or more miscarriages had a more than 50% change of a normal pregnancy on the placebo arm of the study. Similarly, the PRISM study looking at progesterone supplementation in women with threatened miscarriage, until 16 weeks of gestation, also found no evidence of efficacy (Coomarasamy *et al.* 2019). However a detailed analysis of the evidence suggests that if the woman has had a previous miscarriage or previous miscarriages that there was evidence of benefit from progesterone supplementation from onset of the bleeding (Coomarasamy *et al.* 2020).

What might be the effect of prolonged progesterone supplementation in women with threatened miscarriage where there is no evidence of suboptimal progesterone concentrations? It is likely that this is a pharmacological effect rather than a physiological replacement. Progesterone has anti-inflammatory effects, it is immune modulating and it causes quiescence of the myometrium (Shah *et al.* 2019). It could be postulated that some women have an inflammatory or immune defect that predisposes to bleeding in early pregnancy and related increased uterine activity in response to the bleeding. Progesterone in supra-physiological concentrations may help alleviate this in some cases. This positive finding is likely to cause a major shift in how we manage women with threatened miscarriage and the expectation of women with early pregnancy bleeding.

A common argument is that if it might benefit some women why not give it to all women with threatened or recurrent miscarriage. The safety data for progesterone in early pregnancy has been largely reassuring (Piette 2020). There have been reports of an association of progesterone treatment in early pregnancy with developmental abnormalities such as hypospadias (Carmichael *et al.* 2005). As this was not seen in the recent studies (Coomarasamy *et al.* 2020) it might be an association with synthetic gestagen used in the past rather than natural progesterone used currently. There is also some evidence in humans for a link between progesterone exposure and the development of autism spectrum disorder (ASD) in later life (Davidovitch *et al.* 2018). Using a national registry of male births Davidovitch showed that IVF treatment was not associated with ASD. However, progesterone treatment in early pregnancy was associated with ASD (RR 1.51: CI 1.22–1.86, *P* < 0.001).

Although correlation cannot lead to conclusions about causation, this is effect is biologically plausible as foetal steroids regulate epigenetic modulation of the brain, which has been postulated to be involved in the development of ASD (Baron-Cohen *et al.* 2015). Studies on the sheep foetus, at the equivalent of 15 weeks of human gestation, after maternal administration of natural progesterone showed elevated concentrations in male but not female foetuses (Siemienowicz *et al.* 2020). This was associated with functional changes in the pituitary gland and testes and an increase in circulating 11-dehydrocorticosterone, a steroid with mineralocorticoid effects (Siemienowicz *et al.* 2020). Whether this translates into subtle changes in the male offspring phenotype is not known. However, it is well known that foetal exposure to an altered steroid environment has critical roles in sexual differentiation and the programming of health and disease in later life (Ho *et al.* 2017).

True luteal inadequacy does exist and it is iatrogenic in nature and there is a role for physiological progesterone supplementation in assisted conception. There also is a role for pharmacological progesterone supplementation in threatened miscarriage in women with previous miscarriages. However, we cannot say for certain that prolonged foetal exposure to increased progesterone beyond the luteoplacental shift has no effects on the adult offspring. This suggests that we should be cautious about the prolonged use of progesterone supplementation in early pregnancy outside the current guidelines or evidence base.

## Declaration of interest

The author declares that there is no conflict of interest that could be perceived as prejudicing the impartiality of this commentary.
